# Ischaemic Heart Disease and Occupational Exposures: A Longitudinal Linkage Study in the General and Māori Populations of New Zealand

**DOI:** 10.1093/annweh/wxab087

**Published:** 2021-10-09

**Authors:** Lucy A Barnes, Amanda Eng, Marine Corbin, Hayley J Denison, Andrea ‘t Mannetje, Stephen Haslett, Dave McLean, Lis Ellison-Loschmann, Rod Jackson, Jeroen Douwes

**Affiliations:** Centre for Public Health Research, Massey University, Wellington, New Zealand; Centre for Public Health Research, Massey University, Wellington, New Zealand; Centre for Public Health Research, Massey University, Wellington, New Zealand; Centre for Public Health Research, Massey University, Wellington, New Zealand; Centre for Public Health Research, Massey University, Wellington, New Zealand; Centre for Public Health Research, Massey University, Wellington, New Zealand; School of Fundamental Sciences – Statistics, College of Sciences, Massey University, Palmerston North, New Zealand; Statistical Consulting Unit, The Australian National University, Acton Australian Capital Territory, Canberra, Australia; Faculty of Engineering and Information Sciences, University of Wollongong, New South Wales, Australia; Centre for Public Health Research, Massey University, Wellington, New Zealand; Centre for Public Health Research, Massey University, Wellington, New Zealand; Health Services Research Centre, Victoria University of Wellington, Wellington, New Zealand; Section of Epidemiology and Biostatistics, School of Population Health, Faculty of Medical and Health Sciences, The University of Auckland, Auckland, New Zealand; Centre for Public Health Research, Massey University, Wellington, New Zealand

**Keywords:** cardiovascular disease, data linkage, ischaemic heart disease, longitudinal, occupational exposures

## Abstract

**Objectives:**

This study assessed associations between occupational exposures and ischaemic heart disease (IHD) for males and females in the general and Māori populations (indigenous people of New Zealand).

**Methods:**

Two surveys of the general adult [New Zealand Workforce Survey (NZWS); 2004–2006; *n* = 3003] and Māori population (Māori NZWS; 2009–2010; *n* = 2107), with information on occupational exposures, were linked with administrative health data and followed-up until December 2018. Cox proportional hazards regression (adjusted for age, deprivation, and smoking) was used to assess associations between organizational factors, stress, and dust, chemical and physical exposures, and IHD.

**Results:**

Dust [hazard ratio (HR) 1.6, 95%CI 1.1–2.4], smoke or fumes (HR 1.5, 1.0–2.3), and oils and solvents (HR 1.5, 1.0–2.3) were associated with IHD in NZWS males. A high frequency of awkward or tiring hand positions was associated with IHD in both males and females of the NZWS (HRs 1.8, 1.1–2.8 and 2.4, 1.1–5.0, respectively). Repetitive tasks and working at very high speed were associated with IHD among NZWS females (HRs 3.4, 1.1–10.4 and 2.6, 1.2–5.5, respectively). Māori NZWS females working with vibrating tools and those exposed to a high frequency of loud noise were more likely to experience IHD (HRs 2.3, 1.1–4.8 and 2.1, 1.0–4.4, respectively). Exposure to multiple dust and chemical factors was associated with IHD in the NZWS males, as was exposure to multiple physical factors in males and females of the NZWS.

**Conclusions:**

Exposures associated with an elevated IHD risk included dust, smoke or fumes, oils and solvents, awkward grip or hand movements, carrying out repetitive tasks, working at very high speed, loud noise, and working with tools that vibrate. Results were not consistently observed for males and females and between the general and Māori populations.

**What’s Important About This Paper?**

A range of psychosocial, organizational, and environmental workplace factors have been linked to cardiovascular disease; however, ethnic minorities and females are often under-represented in this research. In this study, a number of exposures were associated with ischaemic heart disease (IHD), and multiple occupational exposures increased the risk of incident IHD. Associations were not consistent between males and females or for the Māori and general populations. These findings suggest that occupational risk factors for IHD are not equivalent across all populations and future research and interventions may not be generalizable across all populations.

## Introduction

Growing evidence suggests a role for psychosocial, organizational, and environmental workplace factors in cardiovascular disease (CVD) (Hwang and Hong, 2012). In particular, work-related psychosocial factors, such as stress, have been linked to CVD with relative risk estimates ranging from 1.5 to 1.9 (Sara *et al.*, 2018), and there is suggestive evidence for modest associations with loud noise and shift work. A meta-analysis of a small number of prospective studies on noise estimated an overall relative risk for CVD of 1.34 (95% CI 1.15–1.56) (Skogstad *et al.*, 2016). Organizational factors, such as shift work, have also been studied, with a 2018 meta-analysis suggesting an almost 20% higher risk of CVD for those involved in shift work (Torquati *et al.*, 2018), and another recent meta-analysis demonstrating a dose–response relationship (Cheng *et al.*, 2019). Physical exertion, sedentary behaviour, long working hours, and chemicals (such as pesticides) are among other occupational exposures associated with CVD. However, the extent of research into occupational risk factors for CVD and the consistency of findings has been variable (Kristensen, 1989a,b; Hwang and Hong, 2012).

Most research has been undertaken in males or male-dominated occupations, with effects on female cardiovascular health largely unexplored. Ethnic minorities have also rarely been studied, despite considerable differences in CVD burden and occupational exposure profiles between ethnic groups and males and females (Eng *et al.*, 2011; Denison *et al.*, 2018; Ministry of Health, 2019). Therefore, our understanding of occupational risk factors may not equally apply to all demographic groups, with interventions potentially increasing (rather than decreasing) health inequities. In New Zealand (NZ), Māori (the indigenous population) have a considerably greater CVD burden compared to NZ Europeans (Ministry of Health, 2019), and they are also overrepresented in blue-collar occupations (Milne *et al.*, 2019). The same is true for other indigenous populations (Liebler, 2018; Wiemers *et al.*, 2018) but, to our knowledge, no previous occupational CVD studies have focused on indigenous workers.

In this study we used information from two NZ Workforce Surveys (NZWS), one conducted among Māori and one in the general population, to assess associations between occupational exposures and ischaemic heart disease (IHD), which makes up the greatest proportion of CVD cases, by linkage to routinely collected health records. To allow ethnic and sex-specific associations to be studied, analyses were stratified by survey and sex.

## Methods

This is a longitudinal study using occupational history and lifestyle information from two previously conducted occupational surveys in the general and Māori population (see below). Incident IHD events were identified using routinely collected health data for a 7–14 year period from the date of the interview until 31 December 2018.

### Workforce surveys

The methods for the two New Zealand Workforce Surveys (general population NZWS (Eng *et al.*, 2010) and Māori NZWS (Denison *et al.*, 2018)) have been described in detail previously. Briefly, for each survey, a random proportionally stratified, systematic, and self-weighted sample of people aged 20–64 years were selected from the Māori and general electoral rolls. The general population NZWS was conducted from 2004 to 2006. Invitations to participate in a telephone interview were mailed up to three times and nonresponders were contacted by phone (if available), with 3003 participants (37%) completing the survey. The Māori NZWS was conducted from 2009 to 2010 using the same methodology and resulted in 2107 participants (29%) completing the survey (Denison *et al.*, 2018). Potential for participation bias from low survey response was evaluated and considered to be small (‘t Mannetje *et al.*, 2011), i.e., while some groups were under-represented, the prevalence of key survey variables (both occupational exposure and health-related variables) were unchanged after standardizing to the demographic distribution of the source population, and similar between early and late responders. Two participants were included in both surveys and we, therefore, excluded their most recent interview (i.e., the Māori NZWS).

The questionnaire included questions about lifetime work history, current workplace exposures, and demographic and lifestyle factors. Ethics approval was granted by the Massey University Human Ethics Committee (NZWS—WGTN 03/133, Māori NZWS—MUHEC 08/28) and from the New Zealand Health and Disability Ethics Committee for the linkage of the two surveys (16/NTB/173).

### Self-reported occupational exposures

Participants were asked whether the following exposures were present (yes/no) in their current or most recent workplace environment: dust; smoke or fumes; gas; oil and solvents; acids or alkalis; and pesticides. They were also asked about organizational factors, including working irregular hours (outside 7:00–20:30) and night shift (for at least 3 h between 00:00 and 5:00) in the previous month, as well as the number of hours worked per week (<35, 35–45, 46–54, and ≥55 h). Participants were asked how often their job involved exposure to the following physical factors: awkward and tiring positions; awkward grip or hand movements; lifting; standing; sitting; using tools that vibrate; loud noise; repetitive tasks; working at very high speed; and working to tight deadlines. This was measured on a scale from never to always (provided as a percentage of time or a point on the scale from never, ¼, ½, ¾, or all the time) and the median frequency of exposure (averaged across the cohorts), was calculated to determine a cut-point for ‘low exposure’ (i.e., <=median) and ‘high exposure’ (i.e., >median). Finally, participants rated how stressful they found their current job: none; mild; moderate; very; and extremely stressful, and this was categorized into: none/mild; moderate; and very/extremely.

The occupational exposures included in the analyses were selected based on previously reported associations with CVD (Hwang and Hong, 2012). We also asked about other exposures (e.g. boring work and working outside), but these were not included as they have not previously been shown to be associated with CVD.

### Other CVD risk factors assessed via questionnaire

Participants provided a current or most recent job title with a description of job tasks, and each job was coded using the NZ Standard Classification of Occupations (NZSCO) 1999, which is a hierarchical skills-based classification with nine major occupational groups (Statistics New Zealand, 2001). Age at the interview was categorized as follows: 20–34, 35–44, 45–54, and ≥55 years. Socioeconomic status (SES) was assessed using the NZ Deprivation Index 2006, a census-based index with a relative deprivation score from 1 to 10, based on place of residence. The distribution of deprivation is presented in quintiles, but for subsequent analyses, it was dichotomized combining scores 1–8 (least deprived) and 9–10 (most deprived).

Smoking status at the time of the interview was analysed as never/ever and as pack-years, calculated from the number of cigarettes smoked per day divided by 20, multiplied by the number of years smoked. Results for both measures were very similar (not shown); therefore, only results for ever/never are presented. Body Mass Index (BMI) at the time of interview was calculated using self-reported height and weight grouped into four categories (<18.5, 18.5–24.9, 25–29.9, and ≥30) based on WHO guidelines (World Health Organization, 2000).

### IHD identified from linked health data

IHD events from the deidentified survey information were linked to the Integrated Data Infrastructure (IDI), a longitudinal meta-dataset of de-identified data from government agencies, at the individual level (Milne *et al.*, 2019). Before linkage, probabilistic matching was conducted based on the date of birth, sex, family name, and first two given names, to identify National Health Index numbers to enable linkage to Ministry of Health (MoH) datasets. From the surveys, 98% of respondents were successfully matched and could be linked to mortality, public hospital diagnoses, and pharmaceutical dispensing records.

The IHD definition included IHD deaths, hospital discharges, and procedures using International Classification of Disease (ICD) codes and ≥2 dispensing of anti-anginal drugs from the pharmaceutical claims dataset (see Supplementary Table S1, available at *Annals of Occupational Hygiene* online) and was based on a previously developed definition (Wells *et al.*, 2017). Primary health care information was not used as it is not available in the IDI.

### Follow-up of IHD events

Participants with prior IHD were excluded using the same IHD definition. For incident IHD, participants were followed from the date of interview: 2004–2006 for the general population and 2009–2011 for Māori. Participants that moved overseas or died from other causes were identified through immigration and mortality data in the IDI, respectively, and were censored from that time point. The date last observed for participants that were not lost to follow-up, not deceased, or did not have an IHD event, was 31 December 2018.

### Statistical analyses

Analyses were stratified by survey and sex. Cox proportional hazards regression was used to calculate hazard ratios (HR) for associations between occupational exposures in participants’ current or most recent job, and incident IHD. Models were adjusted for age groups, deprivation, and smoking status. For physical exposures, the three-level variable (none/low-/high-frequency exposure) was used. For analyses where the number of IHD cases was <6 and had to be suppressed due to IDI requirements (see below), both low- and high-level exposures were combined to create a dichotomous variable of exposed versus not exposed.

Associations for combined workplace exposures were assessed by combining dust/chemical exposure variables if they were significantly (*p* < 0.05) and positively associated with IHD in at least one cohort, by summing the number of exposures for each participant (0 (reference), 1, 2, or 3). We used the same approach for physical exposures resulting in a summed exposure variable ranging from 0 to 4. The focus for statistical analysis of ordinal exposure variables was on whether exposure indicated increased IHD risk.

In compliance with the IDI confidentiality requirements, all frequencies were rounded to the nearest multiple of three, and percentages were calculated from the rounded counts (hence total numbers of participants in each table vary slightly and do not add to exactly 100%). All statistical tests used the unrounded counts. All counts under six and the HRs from these are suppressed (marked ‘S’ in the tables).

As the study involved multiple comparisons, we compared the number of expected statistically significant (*p* < 0.05) findings (based on chance) with the number of actual observed statistically significant findings. We also assessed whether the difference in expected and observed significant findings was significantly (*p* < 0.05) different overall. To do this, we determined, via the binomial theorem, the probability of *s*_0_ or more successes from a sequence of *k* Bernoulli trials given the probability of success for each test is *p*. This overall probability is:


$$\begin{array}{l} p_{0}=\sum_{s\geq s_{0}}^{k}{}_{k}C_{s}p^{s}{\left(1-p\right)}^{k-s} \end{array}$$

where ${}_{k}C_{s}$ is the number of ways of choosing *s* items from *k.* Here *p* is set to 0.05. Evaluation of this sum is straightforward for any s and *k* and can proceed iteratively because the ratio of the (*s*+1)th to the *s*th term in the expansion is {(k−s)p}/{(s+1)(1−p)}. The procedure is a variation of the multiple comparison adjustment method of Šidák (Sidak, 1967), except that, rather than setting *p*_0_ and solving for *p*, here *p* is set and the corresponding *p*_0_ is determined.

## Results

A total of 70 participants could not be linked to health data and for 15 a date last observed could not be determined. A further 213 participants (83 NZWS and 130 Māori NZWS) with an IHD event before the interview were excluded, resulting in 2875 participants of the NZWS survey and 1935 participants of the Māori NZWS survey for analyses. Mean follow-up for the NZWS and Māori NZWS were 12.1 and 7.5 years, respectively.

### Incident IHD

There were 135 incident IHD cases in the NZWS and 93 in the Māori NZWS. As expected, IHD cases were overrepresented in males, the oldest age group, ever smokers, overweight/obese BMI categories, and high deprivation group (Table 1). IHD cases were also overrepresented in *plant and machine operators and assemblers* and *elementary occupations*.

**Table 1.** Population characteristics.

	NZWS survey				Māori NZWS survey			
	Total *n* = 2874		IHD cases *n* = 135		Total *n* = 1935		IHD cases *n* = 93	
	Males	Females	Males	Females	Males	Females	Males	Females
	1350 (47.0%)	1524 (53.0%)	99 (73.3%)	36 (26.6%)	852 (44.0%)	1083 (56.0%)	51 (54.8%)	42 (45.2%)
	n (%)	n (%)	n (%)	n (%)	n	n (%)	n (%)	n (%)
Age at interview								
20–34	279 (20.7)	333 (21.9)	S	S	159 (18.7)	207 (19.1)	S	S
35–44	324 (24.0)	459 (30.1)	15 (15.2)	6 (16.7)	201 (23.6)	255 (23.5)	S	S
45–54	390 (28.9)	462 (30.3)	30 (30.3)	15 (41.7)	210 (24.6)	273 (25.2)	9 (17.6)	12 (28.6)
55+	354 (26.2)	273 (17.9)	54 (54.5)	15 (41.7)	288 (33.8)	348 (32.1)	39 (76.5)	27 (64.3)
Interview year								
2004	147 (10.9)	198 (13.0)	15 (15.2)	S	N/A	N/A	N/A	N/A
2005	678 (50.2)	738 (48.4)	57 (57.6)	24 (66.7)	N/A	N/A	N/A	N/A
2006	528 (39.1)	588 (38.6)	30 (30.3)	9 (25.0)	N/A	N/A	N/A	N/A
2007	S	S			N/A	N/A	N/A	N/A
2009	N/A	N/A	N/A	N/A	156 (18.3)	207 (19.1)	12 (23.5)	12 (28.6)
2010	N/A	N/A	N/A	N/A	456 (53.5)	585 (54.0)	30 (58.8)	21 (50.0)
2011	N/A	N/A	N/A	N/A	240 (28.2)	288 (26.6)	12 (23.5)	9 (21.4)
Ethnicity								
Pākehā	1080 (80.4)	1206 (79.1)	78 (78.8)	33 (91.7)				
Māori^a^	99 (7.4)	156 (10.2)	12 (12.1)	S	852 (100.0)	1083 (100.0)	51 (100.0)	42 (100.0)
Pacific peoples	21 (1.6)	33 (2.1)	S	S				
Other	144 (10.7)	129 (8.5)	9 (9.1)	S				
*Missing*	S							
Smoking								
Ever	681 (50.6)	738 (48.4)	60 (60.6)	21 (58.3)	513 (60.4)	717 (66.4)	30 (58.8)	30 (71.4)
Current	246 (18.2)	282 (18.5)	27 (27.3)	9 (25.0)	210 (24.6)	327 (30.2)	6 (11.8)	12 (28.6)
*Missing*	S	S			S	S		
Body mass index								
<18.5	S	27 (1.9)	S	S	S	18 (1.9)	S	S
18.5–24.9	465 (35.1)	717 (49.7)	2 (28.1)	12 (36.4)	135 (16.4)	297 (30.8)	S	9 (23.1)
25–29.9	606 (45.7)	429 (29.7)	39 (40.6)	12 (36.4)	318 (38.7)	255 (26.5)	12 (24.5)	12 (30.8)
30+	249 (18.8)	267 (18.5)	30 (31.3)	9 (27.3)	366 (44.5)	387 (40.2)	30 (61.2)	18 (46.2)
*Missing*	21	84	S	S	30	120	S	S
Deprivation Index 2006								
1–2 (least deprived)	390 (28.9)	378 (24.8)	24 (24.2)	9 (25.0)	129 (15.2)	138 (12.7)	S	S
3–4	306 (22.7)	324 (21.3)	21 (21.2)	6 (16.7)	135 (15.9)	156 (14.4)	6 (11.8)	S
5–6	273 (20.2)	354 (23.2)	18 (18.2)	6 (16.7)	147 (17.3)	195 (18.0)	9 (17.6)	S
7–8	222 (16.4)	279 (18.3)	12 (12.1)	9 (25.0)	201 (23.7)	246 (22.7)	12 (23.5)	18 (42.9)
9–10 (most deprived)	162 (12.0)	186 (12.2)	18 (18.2)	6 (16.7)	234 (27.6)	351 (32.4)	21 (41.2)	15 (35.7)
Missing		S			S	S		
1-digit NZSCO occupational group								
1. Legislators, administrators and managers	261 (19.3)	165 (10.8)	15 (15.2)	S	93 (11.0)	123 (11.5)	9 (17.6)	S
2. Professionals	228 (16.9)	387 (25.4)	12 (12.1)	S	102 (12.1)	213 (19.9)	6 (11.8)	6 (14.3)
3. Technicians and assoc. professionals	168 (12.4)	273 (17.9)	12 (12.1)	9 (25.0)	96 (11.3)	189 (17.6)	S	S
4. Clerks	60 (4.4)	273 (17.9)	9 (9.1)	9 (25.0)	42 (5.0)	156 (14.6)	S	S
5. Service and sales workers	81 (6.0)	267 (17.5)	S	S	75 (8.9)	216 (20.2)	S	12 (28.6)
6. Agriculture and fishery workers	120 (8.9)	60 (3.9)	9 (9.1)	S	69 (8.2)	39 (3.6)	S	S
7. Trade workers	225 (16.7)	18 (1.2)	18 (18.2)	S	120 (14.2)	12 (1.1)	S	S
8. Plant and machine operators and assemblers	144 (10.7)	30 (2.0)	15 (15.2)	9 (25.0)	192 (22.7)	57 (5.3)	9 (17.6)	S
9. Elementary occupations	57 (4.2)	54 (3.5)	9 (9.1)	6 (16.7)	63 (7.4)	69 (6.4)	17 (23.5)	6 (14.3)
Missing	S				6	12		S

Following IDI protocols, frequencies have been rounded to the nearest multiple of three and percentages calculated from those rounded counts.

S = suppressed.

^a^Indigenous people of New Zealand.

### Organizational factors, dust and chemicals, and stress

Dust, smoke or fumes, and oils and solvents (Table 2) were associated with IHD after adjusting for age, deprivation, and smoking in males of the NZWS [HR (95%CI): 1.6 (1.1–2.4); 1.5 (1.0–2.3); and 1.5 (1.0–2.3), respectively]. This was not found in the Māori NZWS. There were no associations observed for other chemicals, organizational factors, or stress in either survey. The proportion of participants exposed to all three chemical exposures (dust; smoke or fumes; oils and solvents) was similar between surveys but considerably greater in males compared to females (NZWS males 12.2%; Māori NZWS males 12.7%; NZWS females 2.6%; Māori NZWS females 2.5%). In NZWS males, exposure to two dust and chemical factors was significantly associated with IHD [HR 2.3 (1.4–3.8)]; this was not found in the other groups (Table 2).

**Table 2.** Associations between IHD and organizational factors, stress, and dust and chemical exposures.

	NZWS						Māori NZWS					
	Males			Females			Males			Females		
	Total	IHD	HR (95% CI)^a^	Total	IHD	HR (95% CI)^a^	Total	IHD	HR (95% CI)^a^	Total	IHD	HR (95% CI)^a^
	1350	99		1524	36		852	51		1083	42	
Organizational factors												
Hours worked												
<35	147	12	0.9 (0.5–1.7)	654	12	0.7 (0.3–1.3)	114	12	1.1 (0.5–2.3)	399	18	2.0 (1.0–4.2)
35–45	642	48	Ref 1.00	660	18	Ref 1.00	375	24	Ref 1.00	498	12	Ref 1.00
46–54	291	18	0.9 (0.5–1.5)	111	S	S	201	9	0.8 (0.4–1.6)	84	S	S
55+	267	21	0.9 (0.5–1.5)	99	S	S	144	S	S	72	S	S
Irregular hours	381	33	1.4 (0.9–2.1)	264	6	0.9 (0.4–2.2)	279	15	0.8 (0.4–1.4)	246	9	1.1 (0.5–2.2)
Night shift	129	12	1.7 (0.9–3.0)	63	S	S	117	S	S	87	S	S
Stress												
None/mild	486	42	Ref 1.00	642	15	Ref 1.00	405	30	Ref 1.00	489	21	Ref 1.00
Moderate	663	42	0.7 (0.5–1.1)	645	15	1.3 (0.6–2.7)	339	15	0.7 (0.4–1.3)	414	12	0.6 (0.3–1.3)
Very/extremely	198	12	0.6 (0.3–1.1)	234	6	1.7 (0.7–4.4)	105	S	S	165	6	0.9 (0.4–2.2)
Dust and chemical exposures												
Dust	546	48	1.6 (1.1–2.4)*	297	S	S	387	24	1.3 (0.8–2.3)	279	12	1.3 (0.7–2.5)
Smoke or fumes	351	33	1.5 (1.0–2.3)*	171	S	S	261	12	0.6 (0.3–1.1)	135	6	2.0 (0.9–4.4)
Gas	150	12	1.3 (0.7–2.3)	78	S	S	99	6	1.1 (0.5–2.6)	81	S	S
Oil and solvents	405	36	1.5 (1.0–2.3)*	198	S	S	246	12	1.0 (0.5–1.9)	114	6	1.8 (0.7–4.2)
Acids or alkalis	186	15	1.2 (0.7–2.0)	90	S	S	126	S	S	57	S	S
Pesticides	195	9	0.6 (0.3–1.1)	78	S	S	114	12	1.0 (0.6–1.8)	69	S	S
Combined exposures: oils and solvents, smoke or fumes, and dust												
0	630	36	Ref 1.00	1053	30	Ref 1.00	348	21	Ref 1.00	699	24	Ref 1.00
1	306	18	1.0 (0.6–1.8)	321	S	S	219	15	1.2 (0.6–2.3)	252	9	1.3 (0.6–2.7)
2	252	30	2.3 (1.4–3.8)**	114	S	S	177	9	0.9 (0.4–2.1)	99	S	S
3	165	12	1.6 (0.8–3.0)	39	S	S	108	S	S	27	S	S

Following IDI protocols, frequencies have been rounded to the nearest multiple of three and percentages calculated from those rounded counts. The hazard ratios and associated 95% confidence intervals are presented in their raw form and were calculated using the unrounded counts.

S = suppressed.

^a^Adjusted for age group, high deprivation, and smoking status.

* *P* < 0.05; **P < 0.01.

### Physical exposures

High exposure to awkward grip or hand movements was associated with IHD in both males and females of the NZWS [Table 3; males HR 1.8 (1.1–2.8); females HR 2.4 (1.1–5.0)], but this was not found in Māori NZWS. Females in the NZWS (but not Māori NZWS females) also had an increased risk of IHD associated with high-frequency exposure to repetitive tasks [HR 3.4 (1.1–10.4)] and working at very high speed [HR 2.6 (1.2–5.5)].

**Table 3.** Associations between IHD and physical exposures.

	NZWS						Māori NZWS					
	Males			Females			Males			Females		
	Total	IHD	HR (95% CI)^a^	Total	IHD	HR (95% CI)^a^	Total	IHD	HR (95% CI)^a^	Total	IHD	HR (95% CI)^a^
	1350	99		1524	36		852	51		1083	42	
Awkward or tiring positions												
No exposure	345	24	Ref 1.00	411	9	Ref 1.00	159	18	Ref 1.00	291	12	Ref 1.00
Low	582	39	1.1 (0.7–1.8)	606	12	0.9 (0.4–2.2)	345	18	0.5 (0.2–1.0)*	393	12	1.2 (0.5–2.7)
High (≥37.5% of the time)	417	36	1.5 (0.9–2.5)	498	18	1.9 (0.9–4.3)	342	18	0.5 (0.3–1.0)	387	18	1.5 (0.7–3.3)
Awkward grip/hand movements												
No exposure	543	39	Ref 1.00	759	12	Ref 1.00	258	21	Ref 1.00	474	18	Ref 1.00
Low	462	27	1.0 (0.6–1.7)	420	9	1.3 (0.6–3.1)	279	12	0.5 (0.3–1.0)	297	12	1.1 (0.5–2.2)
High (≥12.5% of the time)	339	33	1.8 (1.1–2.8)*	339	15	2.4 (1.1–5.0)*	309	15	0.6 (0.3–1.2)	297	9	1.0 (0.5–2.1)
Repetitive tasks												
No exposure	306	24	Ref 1.00	300	S	Ref 1.00	123	6	Ref 1.00	153	6	Ref 1.00
Low	735	45	0.9 (0.6–1.6)	831	18	2.1 (0.7–6.3)	426	27	1.5 (0.6–3.7)	504	18	0.9 (0.4–2.2)
High (≥75% of the time)	300	30	1.5 (0.9–2.7)	384	12	3.4 (1.1–10.4)*	297	18	1.7 (0.7–4.4)	408	15	1.0 (0.4–2.5)
Working at very high speed												
No exposure	552	45	Ref 1.00	549	12	Ref 1.00	264	21	Ref 1.00	369	18	Ref 1.00
Low	495	30	0.9 (0.6–1.5)	549	6	0.7 (0.3–1.9)	315	18	0.8 (0.4–1.6)	348	9	0.8 (0.4–1.7)
High (≥50% of the time)	294	24	1.2 (0.7–2.0)	417	18	2.6 (1.2–5.5)*	270	12	0.7 (0.4–1.5)	357	9	0.7 (0.3–1.6)
Working to tight deadlines												
No exposure	201	21	Ref 1.00	303	9	Ref 1.00	111	15	Ref 1.00	180	12	Ref 1.00
Low	753	51	0.8 (0.5–1.4)	813	21	0.9 (0.4–2.0)	417	21	0.6 (0.3–1.1)	537	15	0.6 (0.3–1.3)
High (≥75% of the time)	387	27	1.0 (0.6–1.8)	405	9	0.8 (0.3–2.2)	324	15	0.5 (0.3–1.0)	354	12	0.6 (0.3–1.5)
Standing												
No exposure	705	51	Ref 1.00	891	24	Ref 1.00	417	30	Ref 1.00	615	24	Ref 1.00
Low	390	27	0.9 (0.6–1.4)	630	12	0.6 (0.3–1.2)	276	12	0.7 (0.4–1.4)	258	9	0.7 (0.3–1.7)
High (12.5% of the time)	249	18	1.0 (0.6–1.6)				153	9	0.9 (0.4–2.0)	198	9	1.3 (0.6–2.8)
Sitting												
No exposure	288	27	Ref 1.00	354	9	Ref 1.00	225	18	Ref 1.00	285	18	Ref 1.00
Low	645	48	0.8 (0.5–1.3)	600	12	1.0 (0.4–2.5)	399	21	0.7 (0.4–1.4)	447	15	0.5 (0.3–1.0)
High (≥50% of the time)	414	24	0.7 (0.4–1.3)	567	18	2.0 (0.9–4.7)	222	15	0.9 (0.4–1.9)	339	6	0.3 (0.1–0.8)*
Lifting												
No exposure	444	33	Ref 1.00	639	15	Ref 1.00	198	15	Ref 1.00	396	15	Ref 1.00
Low	582	39	1.0 (0.6–1.6)	618	12	0.7 (0.3–1.6)	312	18	0.8 (0.4–1.6)	363	12	1.0 (0.5–2.1)
High (≥37.5% of the time)	321	27	1.3 (0.8–2.2)	267	9	1.5 (0.7–3.4)	339	18	0.8 (0.4–1.5)	312	12	1.3 (0.6–2.7)
Tools that vibrate												
No exposure	915	63	Ref 1.00	1359	33	Ref 1.00	438	33	Ref 1.00	894	30	Ref 1.00
Low	279	27	1.4 (0.9–2.2)	159	S	S	225	9	0.6 (0.3–1.2)	177	9	2.1 (1.0–4.4)*
High (≥12.5% of the time)	147	9	1.2 (0.6–2.4)				183	12	1.0 (0.5–2.0)			
Loud noise												
No exposure	585	42	Ref 1.00	1077	27	Ref 1.00	264	24	Ref 1.00	606	18	Ref 1.00
Low	378	30	1.1 (0.7–1.8)	441	12	1.4 (0.7–2.7)	234	9	0.4 (0.2–0.9)*	240	9	1.6 (0.8–3.6)
High (≥37.5% of the time)	387	27	1.2 (0.7–1.9)				345	18	0.7 (0.4–1.3)	225	12	2.3 (1.1–4.8)*
Combined exposures: high-frequency exposure to awkward grip or hand movements, repetitive tasks, working at very high speed and loud noise.												
0	612	42	Ref 1.00	714	9	Ref 1.00	258	21	Ref 1.00	408	18	Ref 1.00
1	360	24	1.1 (0.7–1.8)	465	12	2.2 (0.9–5.2)	231	12	0.7 (0.3–1.5)	297	9	0.7 (0.3–1.6)
2	219	15	1.3 (0.7–2.3)	213	9	4.6 (1.9–11.1)**	165	6	0.6 (0.2–1.5)	189	6	0.8 (0.3–1.9)
3	117	6	1.1 (0.5–2.4)	96	S	S	117	12	1.8 (0.9–3.9)	114	S	S
4	42	9	3.7 (1.8–7.7)**	36	S	S	78	S	S	66	S	S

Following IDI protocols, frequencies have been rounded to the nearest multiple of three and percentages calculated from those rounded counts. The hazard ratios and associated 95% confidence intervals are presented in their raw form and were calculated using the unrounded counts. (S = suppressed).

^a^Adjusted for age group, high deprivation, and smoking status.

* *P* < 0.05; **P < 0.01.

An inverse association with IHD was found for exposure to awkward or tiring positions and loud noise in Māori NZWS males, statistically significant only for the low exposure categories (HRs 0.5 (0.2–1.0) and 0.4 (0.2–0.9), respectively). Among Māori NZWS females, there was a positive association with high exposure to loud noise [HR 2.3 (1.1–4.8)]; exposure to tools that vibrate was also positively associated [HR 2.1 (1.0–4.4)]. Sitting was inversely associated with IHD among female Māori NZWS (but not NZWS females), significant for the high exposure category [HR 0.3 (0.1–0.8)].

The proportion of participants exposed to all four physical exposures that were positively associated with IHD in at least one cohort (high frequency of awkward grip or hand movements; repetitive tasks; working at very high speed; loud noise) was higher among Māori NZWS compared to the NZWS (NZWS males 3.1%; Māori NZWS males 9.2%; NZWS females 2.4%; Māori NZWS females 6.1%). In NZWS males, exposure to three of these physical factors was associated with an almost four times greater IHD risk and in NZWS females, exposure to two physical factors was associated with a 4.6 times greater risk (Table 3). No associations were found for the Māori NZWS.

Physical exposure analyses were repeated using a dichotomous cut-off representing exposure occurring ≥25% of the time (rather than using a median cut-off), which showed very similar results (see Supplementary Table S2, available at *Annals of Occupational Hygiene* online).

## Discussion

Several occupational exposures were associated with incident IHD. Oils and solvents, dust, and smoke or fumes were associated with an increased risk in the general population of males, and a high frequency of awkward grip or hand movements was associated with an increased risk in both males and females of the general population. High frequency of repetitive tasks and working at very high speed were positively associated with IHD among general population females. Among Māori NZWS females, working with tools that vibrate and exposure to loud noise were associated with an increased risk of IHD. No associations for physical factors were observed in Māori NZWS males or females, and none of the occupational exposures were associated with IHD in Māori NZWS males.

There are several explanations that may contribute to the differences in associations observed between Māori and the general population and males and females. First, statistical power was lower in the Māori cohort due to fewer participants and shorter follow-up, resulting in fewer incident IHD events, even though exposure prevalence of physical factors was higher for Māori NZWS males. Similarly, lower exposure prevalence and fewer IHD cases may have contributed to fewer significant associations observed for women. Reduced power was also an issue when comparing different exposure levels, with fewer study participants in the high exposure groups potentially resulting in non-significant findings for high exposure groups even if results for lower exposures were statistically significant.

Second, the nature, as well as circumstance of exposure may differ between groups. This may, for example, have contributed to the effect observed for sitting, which was inversely associated with IHD only in Māori NZWS females. Similarly, ‘tools that vibrate’ used by women can be different from those used by men, which may explain the difference in IHD risk observed between men and women reporting this exposure. More generally, measures of self-reported exposures are relatively crude, which may lead to exposure misclassification, particularly for factors such as ‘working at very high speed’ that, although frequently used as a measure of job intensity in working conditions surveys, are poorly defined and/or quantified. Also, perception and reporting of exposure may differ between Māori and non-Māori and males and females, and exposure misclassification may also differ between these groups, although our analyses, which were stratified by gender and ethnicity, would be less affected.

Thirdly, the distribution of occupations in Māori differs from that of the general population (Table 1), which impacts both exposure prevalence and the occupational composition of the reference group and the interpretation of HRs relative to each survey’s reference group. Adjustment for 1-digit occupational group did not affect results (data not shown), although this adjustment alone may not be sufficient to address this issue, particularly in stratified analyses as reported here. Furthermore, occupational exposures were only available for the current or most recent job, and although the average number of jobs in males and females was similar (NZWS males 4.4; NZWS females 4.9; Māori NZWS males 4.5; Māori NZWS females 4.9), the average duration of employment of the last job was longer for males (NZWS males 9.5. years; NZWS females 6.0 years; Māori NZWS males 9.2 years; Māori NZWS females 6.9 years); exposure estimates may therefore not be entirely comparable for males and females. To partially address these issues, sensitivity analyses were conducted excluding those who were in the job for <5 years (582 NZWS males, 861 NZWS females, 336 Māori NZWS males, 558 Māori NZWS females). Although this resulted in wider confidence intervals and some findings losing statistical significance, particularly for NZWS females, results were largely unchanged (data not shown).

Fourthly, this study included only a limited number of exposures that have previously been associated with IHD. Information on other relevant exposures [e.g., job insecurity, discrimination, electromagnetic fields, physical exertion, and environmental tobacco smoke (Wadsworth *et al.*, 2007; Hwang and Hong, 2012; Virtanen *et al.*, 2013)] was not collected in the original workforce surveys.

In addition to the aforementioned explanations, sex-specific differences in susceptibility and pathophysiology of CVD may play a role in the observed differences between males and females (Shufelt *et al.*, 2018).

We observed an association with dust among general population males, which is consistent with previous studies on occupational particulate matter and CVD (Fang *et al.*, 2010). Similarly, our finding that exposure to smoke or fumes was associated with IHD, is consistent with previous observations that smoke and fumes, such as carbon monoxide and combustion products, were associated with CVD (Kristensen, 1989a; Gustavsson *et al.*, 2001). The evidence is less clear for solvent exposure; earlier work suggested organic solvents may be linked to CVD (Wilcosky and Simonsen, 1991), while a more recent study found no link between solvent exposure and CVD or IHD (Bulka *et al.*, 2019).

In contrast to leisure-time physical activity, occupational physical exertion, and heavy lifting have been linked to increased IHD risk (Petersen *et al.*, 2012; Li *et al.*, 2013; Holtermann *et al.*, 2018). In our study, heavy lifting was not significantly associated with IHD; high frequency of awkward grip or hand movements, on the other hand, was associated with IHD for both sexes, and repetitive tasks and working at very high speed was also associated with IHD for NZWS females. These specific exposures have not previously been studied in the context of IHD and may explain the increased IHD risk observed for occupational groups such as *plant and machine operators and assemblers* (Holmes *et al.*, 2011).

Sedentary behaviour has been associated with CVD (Carter *et al.*, 2017), but there is limited evidence for occupational sedentary behaviour (van Uffelen *et al.*, 2010). In this study, sitting >50% of working time was associated with a reduced risk in female Māori NZWS only. The reasons are unclear, but it is possible that sitting times at work may not adequately represent sedentariness or that it is associated with other work-related risk/protective factors; for example, prolonged sitting may reflect a lack of physically demanding exposures.

Noise has repeatedly been associated with CVD (Skogstad *et al.*, 2016). In this study, frequent exposure to noise was associated with >2 times the risk of IHD in Māori NZWS females, whereas noise exposure in Māori NZWS males was inversely associated. This supports previous literature indicating women may be more adversely affected by noise exposure (Dzhambov and Dimitrova, 2016). In Māori NZWS females, we also found an association with tools that vibrate, and whilst modest evidence of a relationship between vibration and CVD exists, there are limited studies among female workers (Krajnak, 2018).

Chronic stress, including occupational stress, has been associated with CVD (Sara *et al.*, 2018), but this was not observed in this study. It is possible that our measure of stress was insufficiently nuanced, with previous studies using more refined measures, such as job strain and effort-reward imbalance (Sara *et al.*, 2018).

Both shift work and long working hours have previously been linked to IHD, however, the overall evidence is inconsistent (Rivera *et al.*, 2020). In this study, we did not observe significant associations with IHD for the number of hours worked, for working irregular hours, or for working night shifts, but the prevalence of these exposures was low.

Few studies considered the combined effect of occupational exposures, but some analysed exposure interactions (e.g., physical activity, noise, job strain, and shift work), and reported additive effects (Virkkunen *et al.*, 2006; Eriksson *et al.*, 2018). In this study, exposure to multiple occupational factors was associated with greater IHD risk, which may explain the elevated prevalence of IHD risk factors we have previously observed for some occupational groups (e.g. *plant and machine operators and assemblers*; *elementary occupations* (Barnes *et al.*, 2020)) where exposure to multiple risk factors is common.

Limitations, in addition to those described above, include that we did not have access to private hospital or primary health care information, which may have resulted in an underestimation of incident IHD, although we did have access to community dispensing of anti-angina medications, which likely captured most IHD cases that did not result in public hospitalization. Community dispensing data had limited date range availability (2005 onward); however, most incident IHD events were identified through hospital admissions (73–80% across males and females of both surveys), which were available from 1988 onward. A related issue is that due to more limited access to tertiary hospitals in rural areas IHD diagnoses may be undercounted in these areas. However, the use of community dispensing of anti-angina medications in our IHD definition will likely have captured most cases that did not result in public hospitalization. In addition, the percentage of New Zealanders living in rural areas is relatively small [16.3% and 18.0% for the general and Māori population, respectively (Environmental Health Intelligence New Zealand, 2020)]. Any potential bias resulting from undercounting IHD cases in rural areas would therefore be minor.

There may be confounders that were not considered, such as diet, leisure-time physical activity, and alcohol consumption. However, analyses adjusting for BMI did not significantly alter results other than slightly change *p*-values (data not shown). We did not adjust analyses for high blood pressure, diabetes, elevated cholesterol as these may be on the causal pathway between (some) occupational exposures and IHD.

Finally, assessing multiple exposures stratified by sex and survey resulted in a large number of comparisons, so some statistically significant results may be due to chance. However, as shown in Supplementary Table S3, available at *Annals of Occupational Hygiene* online, we found more significant results than would occur by chance. This was particularly the case for analyses involving males and females of the NZWS (Tables 2 and 3) and analyses described in Supplementary tables (Table S2, available at *Annals of Occupational Hygiene* online) for females in the Māori NZWS. For the remainder of the analyses, we still found more statistically significant findings than expected by chance, but the differences were less pronounced and findings of these analyses should therefore be interpreted with caution, particularly for those not previously reported in the literature, and for subgroup analyses with a small number of significant findings.

Although there are limitations of this study, there are also major strengths including the large proportion of females (>50%) and Māori (40%), the inclusion of a range of exposures, which were collected before IHD diagnosis, limiting recall bias, as well as the measure of IHD incidence not relying on self-reports.

In conclusion, associations with exposure to dust, smoke or fumes, oils and solvents, awkward grip or hand movements, carrying out repetitive tasks, working at very high speed, loud noise, and working with tools that vibrate and IHD were found, but results were often not consistent for males and females and between the general and Māori populations. These findings suggest that occupational risk factors for IHD may differ across populations.

## Supplementary Material

wxab087_suppl_Supplementary_Tables

## Data Availability

No data are available due to the confidentiality requirements of the Integrated Data Infrastructure.

## Disclaimer

The results in this research article are not official statistics. They have been created for research purposes from the Integrated Data Infrastructure (IDI), managed by Statistics New Zealand.

The opinions, findings, recommendations, and conclusions expressed in this paper are those of the author(s), not Statistics NZ.

Access to the anonymized data used in this study was provided by Statistics NZ under the security and confidentiality provisions of the Statistics Act 1975. Only people authorized by the Statistics Act 1975 are allowed to see data about a particular person, household, business, or organization, and the results in this paper have been confidentialized to protect these groups from identification and to keep their data safe.

Careful consideration has been given to the privacy, security, and confidentiality issues associated with using administrative and survey data in the IDI. Further detail can be found in the Privacy impact assessment for the Integrated Data Infrastructure available from www.stats.govt.nz.

## References

[CIT0001] Barnes LA, Eng A, Corbin M et al (2020) The prevalence of cardiovascular risk factors in different occupational groups in New Zealand. Ann Work Expo Health; 64: 645–58. 32318690 10.1093/annweh/wxaa040

[CIT0002] Bulka CM, Daviglus ML, Persky VW et al (2019) Association of occupational exposures with cardiovascular disease among US Hispanics/Latinos. Heart; 105: 439–48. 30538094 10.1136/heartjnl-2018-313463 PMC6580877

[CIT0003] Carter S, Hartman Y, Holder S et al (2017) Sedentary behavior and cardiovascular disease risk: mediating mechanisms. Exerc Sport Sci Rev; 45: 80–6. 28118158 10.1249/JES.0000000000000106

[CIT0004] Cheng M, He H, Wang D et al (2019) Shift work and ischaemic heart disease: meta-analysis and dose-response relationship. Occup Med (Lond); 69: 182–8. 30923828 10.1093/occmed/kqz020

[CIT0005] Denison HJ, Eng A, Barnes LA et al (2018) Inequities in exposure to occupational risk factors between Māori and non-Māori workers in Aotearoa New Zealand. J Epidemiol Commun Health; 72: 809–16. 10.1136/jech-2018-210438 29720390

[CIT0006] Dzhambov AM, Dimitrova DD. (2016) Occupational noise and ischemic heart disease: a systematic review. Noise Health; 18: 167–77. 27569404 10.4103/1463-1741.189241 PMC5187658

[CIT0007] Eng A, ‘T Mannetje A, Cheng S et al (2010) The New Zealand workforce survey I: self-reported occupational exposures. Ann Occup Hyg; 54: 144–53. 20080811 10.1093/annhyg/mep097

[CIT0008] Eng A, ‘t Mannetje A, McLean D et al (2011) Gender differences in occupational exposure patterns. Occup Environ Med; 68: 888–94. 21486991 10.1136/oem.2010.064097

[CIT0009] Environmental Health Intelligence New Zealand. (2020) Urban-rural profile. Wellington: Environmental Health Intelligence New Zealand. Available at https://www.ehinz.ac.nz/indicators/population-vulnerability/urbanrural-profile/ Accessed 1 July 2021.

[CIT0010] Eriksson HP, Andersson E, Schiöler L et al (2018) Longitudinal study of occupational noise exposure and joint effects with job strain and risk for coronary heart disease and stroke in Swedish men. BMJ Open; 8: e019160. 10.1136/bmjopen-2017-019160 PMC5892764 29615446

[CIT0011] Fang SC, Cassidy A, Christiani DC. (2010) A systematic review of occupational exposure to particulate matter and cardiovascular disease. Int J Environ Res Public Health; 7: 1773–806. 20617059 10.3390/ijerph7041773 PMC2872342

[CIT0012] Gustavsson P, Plato N, Hallqvist J et al (2001) A population-based case-referent study of myocardial infarction and occupational exposure to motor exhaust, other combustion products, organic solvents, lead, and dynamite. Stockholm Heart Epidemiology Program (SHEEP) Study Group. Epidemiology; 12: 222–8. 11246584 10.1097/00001648-200103000-00015

[CIT0013] Holmes E, Davies A, Wright C et al (2011) Mortality rates according to occupation in New Zealand males: 2001-2005. N Z Med J; 124: 16–28. 21475336

[CIT0014] Holtermann A, Krause N, van der Beek AJ et al (2018) The physical activity paradox: six reasons why occupational physical activity (OPA) does not confer the cardiovascular health benefits that leisure time physical activity does. Br J Sports Med; 52: 149–50. 28798040 10.1136/bjsports-2017-097965

[CIT0015] Hwang WJ, Hong O. (2012) Work-related cardiovascular disease risk factors using a socioecological approach: implications for practice and research. Eur J Cardiovasc Nurs; 11: 114–26. 22357786 10.1177/1474515111430890

[CIT0016] Krajnak K . (2018) Health effects associated with occupational exposure to hand-arm or whole body vibration. J Toxicol Environ Health B Crit Rev; 21: 320–34. 30583715 10.1080/10937404.2018.1557576 PMC6415671

[CIT0017] Kristensen TS. (1989a) Cardiovascular diseases and the work environment. A critical review of the epidemiologic literature on chemical factors. Scand J Work Environ Health; 15: 245–64. 2672297 10.5271/sjweh.1854

[CIT0018] Kristensen TS. (1989b) Cardiovascular diseases and the work environment. A critical review of the epidemiologic literature on nonchemical factors. Scand J Work Environ Health; 15: 165–79. 2675305 10.5271/sjweh.1864

[CIT0019] Li J, Loerbroks A, Angerer P. (2013) Physical activity and risk of cardiovascular disease: what does the new epidemiological evidence show? Curr Opin Cardiol; 28: 575–83. 23928923 10.1097/HCO.0b013e328364289c

[CIT0020] Liebler C . (2018) Occupational dissimilarity between the American Indian/Alaska native workforce and white workforce in the contemporary United States. Am Indian Cult Res J; 42: 41–70. 30899126 10.17953/aicrj.42.1.liebler PMC6424522

[CIT0022] Milne BJ, Atkinson J, Blakely T, et al (2019) Data resource profile: the New Zealand integrated data ifrastructure (IDI). Int J Epidemiol; 48: 677–77e. 30793742 10.1093/ije/dyz014

[CIT0023] Ministry of Health. (2019) Mortality 2017 data tables. Wellington: Ministry of Health.

[CIT0024] Petersen CB, Eriksen L, Tolstrup JS et al (2012) Occupational heavy lifting and risk of ischemic heart disease and all-cause mortality. BMC Public Health; 12: 1070. 23231790 10.1186/1471-2458-12-1070 PMC3538157

[CIT0025] Rivera AS, Akanbi M, O’Dwyer LC et al (2020) Shift work and long work hours and their association with chronic health conditions: a systematic review of systematic reviews with meta-analyses. PLoS One; 15: e0231037. 32240254 10.1371/journal.pone.0231037 PMC7117719

[CIT0026] Sara JD, Prasad M, Eleid MF, et al (2018) Association between work-related stress and coronary heart disease: a review of prospective studies through the job strain, effort-reward balance, and organizational justice models. J Am Heart Assoc; 7: e008073. 29703810 10.1161/JAHA.117.008073 PMC6015274

[CIT0027] Shufelt CL, Pacheco C, Tweet MS et al (2018) Sex-Specific physiology and cardiovascular disease. Adv Exp Med Biol; 1065: 433–54. 30051400 10.1007/978-3-319-77932-4_27 PMC6768431

[CIT0028] Sidak Z . (1967) Rectangular confidence regions for the means of multivariate normal distributions. J Am Stat Assoc; 62: 626–33.

[CIT0029] Skogstad M, Johannessen HA, Tynes T et al (2016) Systematic review of the cardiovascular effects of occupational noise. Occup Med (Lond); 66: 10–6. 26732793 10.1093/occmed/kqv148

[CIT0030] Statistics New Zealand. (2001) New Zealand standard classification of occupations, 1999. Wellington: Statistics New Zealand.

[CIT0021] ‘t Mannetje A, Eng A, Douwes J et al (2011) Determinants of non-response in an occupational exposure and health survey in New Zealand. Aust N Z J Public Health; 35: 256–63. 21627726 10.1111/j.1753-6405.2011.00703.x

[CIT0031] Torquati L, Mielke GI, Brown WJ et al (2018) Shift work and the risk of cardiovascular disease. A systematic review and meta-analysis including dose-response relationship. Scand J Work Environ Health; 44: 229–38. 29247501 10.5271/sjweh.3700

[CIT0032] van Uffelen JG, Wong J, Chau JY et al (2010) Occupational sitting and health risks: a systematic review. Am J Prev Med; 39: 379–88. 20837291 10.1016/j.amepre.2010.05.024

[CIT0033] Virkkunen H, Härmä M, Kauppinen T et al (2006) The triad of shift work, occupational noise, and physical workload and risk of coronary heart disease. Occup Environ Med; 63: 378–86. 16709702 10.1136/oem.2005.022558 PMC2078113

[CIT0034] Virtanen M, Nyberg ST, Batty GD et al; IPD-Work Consortium. (2013) Perceived job insecurity as a risk factor for incident coronary heart disease: systematic review and meta-analysis. BMJ; 347: f4746. 23929894 10.1136/bmj.f4746 PMC3738256

[CIT0035] Wadsworth E, Dhillon K, Shaw C et al (2007) Racial discrimination, ethnicity and work stress. Occup Med (Lond); 57: 18–24. 16928781 10.1093/occmed/kql088

[CIT0036] Wells S, Riddell T, Kerr A et al (2017) Cohort profile: the PREDICT Cardiovascular Disease Cohort in New Zealand Primary Care (PREDICT-CVD 19). Int J Epidemiol; 46: 22. 26686841 10.1093/ije/dyv312

[CIT0037] Wiemers PD, Marney L, Yadav S et al (2018) An overview of indigenous Australian disadvantage in terms of Ischaemic Heart Disease. Heart Lung Circ; 27: 1274–84. 29929920 10.1016/j.hlc.2018.03.003

[CIT0038] Wilcosky TC, Simonsen NR. (1991) Solvent exposure and cardiovascular disease. Am J Ind Med; 19: 569–86. 2053576 10.1002/ajim.4700190503

[CIT0039] World Health Organization. (2000) Obesity: preventing and managing the global epidemic. Report of a WHO Consultation on Obesity, Geneva, 1997 (WHO/NUT/NCD/98.1). Geneva: World Health Organization. 11234459

