# Supplementary material for: Ischaemic Heart Disease and Occupational Exposures: A Longitudinal Linkage Study in the General and Māori Populations of New Zealand
Source: Ann Work Expo Health. 2021 Oct 9;66(4):433–46. doi: 10.1093/annweh/wxab087 (PMC9029235; doi:10.1093/annweh/wxab087)
Supplement: wxab087_suppl_Supplementary_Tables [file wxab087_suppl_supplementary_tables.docx]

**Ischaemic heart disease and occupational exposures – a longitudinal linkage study in the general and Māori populations of New Zealand**

Barnes Lucy A^1^, Eng Amanda^1^, Corbin Marine^1^, Denison Hayley J^1^, 't Mannetje Andrea^1^, Haslett Stephen^1,2,3,4^, McLean Dave^1^, Ellison-Loschmann Lis^1,5^, Jackson Rod^6^, Douwes Jeroen^1^

*^1^ Centre for Public Health Research, Massey University, Wellington, New Zealand*

*^2^ School of Fundamental Sciences – Statistics, College of Sciences, Massey University, Palmerston North, New Zealand*

*^3^ Statistical Consulting Unit, The Australian National University, Acton Australian Capital Territory, Canberra, Australia*

*^4^Faculty of Engineering and Information Sciences, University of Wollongong, New South Wales, Australia*

*^5^Health Services Research Centre, Victoria University of Wellington, Wellington, New Zealand*

*^6^ Section of Epidemiology and Biostatistics, School of Population Health, Faculty of Medical and Health Sciences, The University of Auckland, Auckland, New Zealand*

| **Table S1: IHD Definition** | | |
| --- | --- | --- |
| **Data Source** | **ICD Code or Criteria** | **Details** |
| Mortality data (1988-31 Dec 2016)  National Minimum Dataset (NMDS): Publicly funded hospital discharges –diagnosis/procedure information (1988- 31 Dec 2018) | ICD-9-CMA 410, ICD-10-AM I21 | Acute myocardial infarction |
|  | ICD-10-AM I22 | Subsequent myocardial infarction |
|  | ICD-9-CMA 411, ICD-10-AM I24 | Other acute and subacute forms of ischaemic heart disease |
|  | ICD-9-CMA I23 | Certain current complications following myocardial infarction (within the 28-day period) |
|  | ICD-9-CMA 413, ICD-10-AM I20 | Angina pectoris |
|  | ICD-9-CMA 412, ICD-9-CMA 414,  ICD-10-AM I25^*^ | Chronic ischaemic heart disease |
|  | ICD-9-CMA V45.81 | Aortocoronary bypass status |
|  | ICD-9-CMA V45.82 | Percutaneous transluminal coronary angioplasty status |
|  | ICD-10-AM Z95.1 | Presence of aortocornary bypass graft |
|  | ICD-10-AM Z95.5 | Presences of coronary angioplasty implant and graft |
|  | ICD-9- CMA 36.0x, 36.1x  ICD-10-AM 3530400, 3530401, 3530500, 3530501, 3531000, 3531001, 3531002, 3531003, 3531004, 3531005, 3849700, 3849701, 3849702, 3849703, 3849704, 03849705, 3849706, 3849707, 3850000, 3850001, 3850002, 3850003, 3850004, 3850300, 3850301, 3850302, 3850303, 3850304, 9020100, 9020101, 9020102, 9020103, 3863700, 3845619, 3865308, 3850500 | Procedures |
| Pharmaceutical Data (2005-31 Dec 2018) | 1577 Glyceryl trinitrate  2377 Isosorbide dinitrate  2836 Isosorbide mononitrate  1292 Nicorandil  1949 Perhexiline maleate | >2 dispensings within 12 months |
| *ICD-10-AM I25.2 was excluded as it refers to old myocardial infarction and therefore, not an incident IHD | | |

| **Table S2: Association between physical exposures (25% cut-off point) and IHD** | | | | | | | | | | | | | | |
| --- | --- | --- | --- | --- | --- | --- | --- | --- | --- | --- | --- | --- | --- | --- |
|  | **NZWS** | | | | | | **Māori NZWS** | | | | | | | |
|  | **Males** | | | **Females** | | | **Males** | | | | **Females** | | | |
|  | **Total** | **IHD** | **HR (95%CI)^a^** | **Total** | **IHD** | **HR (95%CI)^a^** | **Total** | **IHD** | **HR (95%CI)^a^** | **Total** | | **IHD** | **HR (95%CI)^a^** |  |
|  | 1350 | 99 |  | 1524 | 36 |  | 852 | 51 |  | 1083 | | 42 |  |  |
| **Physical exposures** | | | | | | | | | | | | | | |
| Awkward or tiring positions | 729 | 57 | 1.4 (0.9-2.0) | 882 | 24 | 1.6 (0.8-3.1) | 597 | 30 | 0.7 (0.4-1.2) | 663 | | 27 | 1.4 (0.7-2.7) |  |
| Awkward grip/hand movements | 546 | 48 | 1.6 (1.09-2.4)***** | 555 | 18 | 2.0 (1.0-3.8)***** | 477 | 24 | 0.7 (0.4-1.2) | 465 | | 18 | 0.9 (0.5-1.7) |  |
| Repetitive tasks | 870 | 66 | 1.4 (0.9-2.2) | 1089 | 33 | 2.5 (1.0-6.0)***** | 681 | 45 | 1.7 (0.7-3.7) | 846 | | 27 | 0.8 (0.4-1.6) |  |
| Working at very high speed | 639 | 45 | 1.2 (0.83-1.9) | 834 | 24 | 1.7 (0.8-3.3) | 507 | 27 | 0.9 (0.5-1.6) | 639 | | 18 | 0.7 (0.3-1.3) |  |
| Working to tight deadlines | 1005 | 66 | 1.0 (0.6-1.5) | 1092 | 24 | 1.1 (0.5-2.2) | 690 | 36 | 0.5 (0.3-1.0)***** | 846 | | 24 | 0.7 (0.4-1.5) |  |
| Standing | 363 | 30 | 1.1 (0.7-1.7) | 435 | 9 | 0.9 (0.4-1.8) | 285 | 15 | 0.7 (0.4-1.4) | 318 | | 12 | 1.1 (0.5-2.2) |  |
| Sitting | 861 | 57 | 0.8 (0.5-1.2) | 1002 | 30 | 2.1 (1.0-4.7) | 510 | 30 | 0.9 (0.5-.7) | 693 | | 18 | 0.5 (0.3-0.9)***** |  |
| Lifting | 582 | 42 | 1.1 (0.7-1.7) | 546 | 15 | 1.3 (0.7-2.4) | 531 | 33 | 0.9 (0.5-1.6) | 516 | | 18 | 1.1 (0.8-2.0) |  |
| Tools that vibrate | 231 | 18 | 1.2 (0.7-2.1) | 87 | S | S | 291 | 12 | 0.7 (0.4-1.4) | 117 | | 9 | 3.0 (1.4-6.3)****** |  |
| Loud noise | 543 | 39 | 1.1 (0.7-.1.6) | 309 | 9 | 1.4 (0.7-3.0) | 492 | 27 | 0.8 (0.5-1.4) | 363 | | 18 | 1.9 (1.0-3.6)***** |  |
| *****P value <0.05, ******P value <0.01. | | | | | | | | | | | | | | |
| Following IDI protocols, frequencies have been rounded to the nearest multiple of three and percentages calculated from those rounded counts. The hazard ratios and associated 95% confidence intervals are presented in their raw form and were calculated using the unrounded counts. (S = suppressed) | | | | | | | | | | | | | | |
| ^a^Adjusted for age, high deprivation and smoking status | | | | | | | | | | | | | | |

| **Table S3: The number of expected statistically significant findings (based on chance) versus the number of actual observed statistically significant findings.** | | | | |
| --- | --- | --- | --- | --- |
|  | Total number of tests | Expected number of significant (p=0.05) findings by chance | Observed number of significant (p<0.05) findings | p-value^a^ for the difference between expected and observed number of significant findings |
| **Main tables** | | | | |
| NZWS males | 40 | 2.0 | 6 | 0.014* |
| NZWS females | 22 | 1.1 | 4 | 0.023* |
| Māori NZWS males | 34 | 1.7 | 2 | 0.512 |
| Māori NZWS females | 29 | 1.5 | 3 | 0.175 |
| **Supplementary tables** | | | | |
| NZWS males | 10 | 0.5 | 1 | 0.401 |
| NZWS females | 9 | 0.5 | 2 | 0.071 |
| Māori NZWS males | 10 | 0.5 | 1 | 0.401 |
| Māori NZWS females | 10 | 0.5 | 3 | 0.012* |
| ^a^The probability tabulated is for the actual number of successes (i.e. significant findings) from a sequence of Bernoulli trials when the probability of success for each trial or test is p=0.05 | | | | |
